# Intracisternal injection of two human SOD1 aggregate strains induces templated aggregation and ALS in transgenic mouse model

**DOI:** 10.1186/s40478-026-02473-x

**Published:** 2026-10-27

**Authors:** Isabelle Sigfridsson, Matthew Marklund, Peter Munch Andersen, Per Zetterström, Stefan L. Marklund, Thomas Brännström

**Affiliations:** 1 Department of Medical Biosciences, Umeå University, 901 87 Umeå, Sweden; 2 Department of Clinical Sciences, Neuroscience, Umeå University, 901 87 Umeå, Sweden

**Keywords:** Superoxide dismutase-1, Cerebrospinal fluid, Amyotrophic lateral sclerosis, Prions, Neurodegeneration

## Abstract

Cytosolic aggregates containing misfolded superoxide dismutase-1 (SOD1) protein is an established hallmark of ALS caused by mutations in the *SOD1* gene. We have previously identified two structurally distinct strains of human (h) SOD1 aggregates (denoted A and B) in mice transgenic (Tg) for hSOD1. Strain A and B have both been shown to transmit templated aggregation and induce early-onset of fatal ALS-like disease when injected directly into the spinal cord or peripheral nerve of hSOD1 Tg mice. In contrast, peripheral administration of hSOD1 aggregates into the intraperitoneal cavity or skeletal muscle did not induce disease. Collectively, these studies provide insight on the spreading mechanism of SOD1. However, the complete map of propagation routes and underlying mechanisms utilized by SOD1 aggregates in ALS is not fully charted. Here, we investigated whether hSOD1 aggregates may spread to the central nervous system (CNS) via the cerebrospinal fluid (CSF). Mutant hSOD1 aggregate seeds or control samples prepared from spinal cords of hSOD1 Tg mice or non-Tg mice, respectively, were inoculated into the cisterna magna of adult 100-day-old Tg mice expressing the G85R mutated hSOD1 protein. Disease phenotyping as well as biochemical and immunohistochemical analyses were performed and compared across the experimental groups. The mice inoculated with hSOD1 seeds developed early signs of ALS by 90 days after inoculation and reached terminal stage approximately 180 days earlier than mice receiving control seeds. Additionally, both strain A and B were observed to initiate templated aggregation along the neuroaxis. The results show that hSOD1 aggregates are capable of transmitting from the CSF to the CNS where they induce ALS-like pathology and concomitant phenotype. Unraveling the propagation pathways utilized by SOD1 opens new avenues to assess treatment strategies against ALS.

**Supplementary Information:**

The online version contains supplementary material available at https://doi.org/10.1186/s40478-026-02473-x.

## Introduction

Amyotrophic lateral sclerosis (ALS) is a lethal neurodegenerative disease characterized by adult-onset of progressive loss of upper and lower motor neurons in the cortex, brainstem and spinal cord. Characteristically, ALS begins with focal paresis in one myotome which spreads in a contiguous way to adjacent myotomes eventually resulting in generalized wasting of most skeletal muscles and paresis [1–3]. There is presently no treatment that arrests the disease, and death usually occurs 3–5 years after symptom onset due to paresis of the respiratory muscles.

Superoxide dismutase-1 (*SOD1*) was the first gene found to be associated with ALS [4], and mutations in this gene are found in approximately 12–15% and 1–2% of all familial and sporadic cases, respectively [5, 6]. Mutations in *SOD1* result in protein misfolding, and the histopathological presence of SOD1 aggregates is an established hallmark of SOD1-associated ALS. Instrumental to getting insight into the biology of mutant SOD1-mediated ALS has been the transgenic (Tg) overexpression in rodents of several SOD1 mutations found in human ALS [7–10].

We have previously identified two structurally distinct strains of human SOD1 aggregates, denoted A and B, in Tg mice expressing human (h) *SOD1* mutations [11]. Strain A and B have been shown to transmit templated aggregation and induce early-onset of fatal ALS-like disease when inoculated into asymptomatic hSOD1 Tg mice [12]. These prion-like properties of hSOD1 aggregates have been supported by several studies showing the ability of SOD1 aggregates to transmit further aggregation [13–17]. The observations are similar to those made for other proteins involved in neurodegenerative diseases, such as amyloid-β [18–20], α-synuclein [21–23] and tau [24, 25], which have also shown prion-like characteristics.

While prion-like spread of SOD1 aggregation is established, the intercellular propagation routes of SOD1 in vivo are still not fully charted. Previous studies have shown that inoculation of spinal homogenates from terminal mutant SOD1 transgenic mice into the spinal cord or sciatic nerve results in retrograde spread of ALS pathology along the neuroaxis [12, 26]. These observations suggest misfolded mutant SOD1 to be capable of spreading within the nervous system, e.g. by transsynaptic transmission [12, 26]. In contrast, peripheral administration of even larger amount of hSOD1 aggregates into the intraperitoneal cavity or skeletal muscle did not induce ALS-like disease, suggesting that SOD1 aggregates do not spread via blood circulation or neuromuscular junctions [27].

A further potential pathway is spreading of the disease via the cerebrospinal fluid (CSF) circulation [28–30]. Although CSF serves to clear the brain from waste products through the glymphatic system [31], emerging evidence suggests that CSF may play a role in the intercellular spread of proteinopathies [32–34]. A previous study suggests e.g. that amyloid-β within the CSF compartment may recirculate through the brain along the same glymphatic route as they are normally cleared [32]. In accordance with this finding, a recent study in humans indicates that a significant proportion of the cerebrospinal fluid recirculates back to the cerebrospinal compartment after it has flown into the interstitial fluid compartment [30]. Indeed, misfolded SOD1 protein has been detected in the CSF of ALS patients and ALS Tg rats [35–37]. The concentration of misfolded SOD1 in human CSF is very low [38] (about 1/1300 of total SOD1), precluding further structural assessment. While the presence of misfolded SOD1 in CSF has been demonstrated, it is not yet known whether misfolded SOD1 in the CSF may re-enter the CNS and continue the spread of disease.

To test the hypothesis that CSF constitutes a route for the spread of SOD1-ALS, we performed intracisternal injection of strain A and B SOD1 aggregates in adult asymptomatic hSOD1 Tg mice. Here, we report that mice inoculated with hSOD1 aggregates developed early signs of ALS by 90 days after inoculation and reached terminal stage approximately 180 days earlier than mice receiving control samples. Moreover, both biochemical and histopathological examinations reveal templated aggregation spreading along the neuroaxis initiated by strain A and B seeds. The results show that hSOD1 aggregates can transmit from the CSF to the CNS where they induce ALS.

## Materials & methods

### Mice

In this study, we used transgenic hemizygous mice expressing G85R mutant human SOD1 (hSOD1^G85R^) [7]. The lifespan of this mouse model is 405 ± 54 days (*n* = 287) in our laboratory and there is no difference between the sexes (females: 406 ± 51 days (*n* = 133), males: 404 ± 56 days (*n* = 154)). The transgenic hSOD1^G85R^ mice have a long window of opportunity for seeing increases in aggregation since aggregates of hSOD1^G85R^ first appear shortly before onset of paresis contrasting the early appearance of high molecular weight aggregates in the hSOD1^G93A^ transgenic mice [12]. The hSOD1^G85R^ mouse strain was used as recipients for the inoculations and for the preparation of strain A seeds. End-stage homozygous mice expressing hSOD^D90A^ were used for the preparation of strain B seeds [10]. Both mouse strains were backcrossed over 50 generations and maintained on the C57BL/6 background. Non-transgenic C57BL/6 mice were used for the preparation of control seeds. All procedures involving the mice described in this article were approved by the Umeå Regional Ethics Committee for Animal Research (Umeå, Sweden).

### Antibodies

The antibodies used in this study are in-house produced and cover almost the entire length of the 153 amino acid (aa) hSOD1 sequence. For the binary epitope mapping, eight different polyclonal primary antibodies were used which were raised in rabbits against peptides corresponding to aa 4–20, 24–39, 43–57, 57–72, 80–96, 100–115, 111–127 and 131–153 in the hSOD1 sequence. These antibodies only bind to disordered sequences of unfolded or misfolded SOD1 and not to the natively folded SOD1 protein [11].

For immunohistochemical detection of misfolded hSOD1, the polyclonal antibodies raised in rabbits against hSOD1 aa 131–153 and aa 111–127 were used. The anti-hSOD^111–127^ Ab was used to specifically target strain B aggregates, based on previous structural analysis of hSOD1 strains [11].

### Preparation of spinal cord homogenate and seeds for inoculations

Material for inoculation was produced in two different ways, either as a homogenate or as isolation of a high molecular weight fraction. These methods result in aggregate species which have the same BEM-profile [12]. A homogenate containing hSOD1 strain A aggregates was prepared from spinal cords from terminally ill hemizygous hSOD1^G85R^ Tg mice. The spinal cords were homogenized in 9 volumes of phosphate-buffered saline (PBS) using a Ultraturrax apparatus (IKA) followed by sonication and centrifugation at 1000 x g and 4 °C for 10 min. The resulting supernatant was aliquoted and kept at -80 °C until use for the inoculations. The amount of insoluble SOD1 aggregates present in the homogenate was calculated using ultracentrifugation and quantitative western blot as previously described [38].

To prepare hSOD1 aggregate seeds, a protocol was developed to specifically preserve high molecular weight fractions. Strain A and strain B seeds as well as control samples were prepared from spinal cords from terminally ill hemizygous hSOD1^G85R^, homozygous hSOD1^D90A^ with short lifespan (369 ± 24) and age-matched non-transgenic C57BL/6 mice, respectively. The hSOD1^D90A^ mice express both strain A and B aggregates, but B-aggregates dominate in the mice with short lifespans [11]. The spinal cords were homogenized in 5 volumes of PBS containing 2% NP40, 160 µl/ml Complete EDTA-free protease inhibitor cocktail (Roche) and 1.8 mM EDTA using an Ultraturrax apparatus followed by sonication. The homogenates were further sonicated in 0.25 M guanidinium chloride (GdmCl) and shaken for one hour at 4 °C. The homogenates were diluted in water containing 1% NP40 to reach a physiological ion strength (0.15 M) and sonicated prior to centrifugation at 1000 x g and 4 °C for 20 min. The resulting supernatants were mixed with iohexol to a concentration of 3% and layered on top of iohexol density cushions consisting of one layer 75.5% iohexol and one layer 13% iohexol. The iohexol density cushion was centrifuged at 336,800 x g for 3 h. In an earlier study [16] where centrifugation at 175,000 x g for 1 h were used. Thus, the present protocol should primarily isolate proteinaceous components with molecular weights >1 × 10^6^ kDa [39]. The supernatants were carefully removed, and dialysis was performed on the bottom layer of the density cushion using Float-a-lyzer G2 MWCO 20 kDa (Pierce) and PBS. The solution was concentrated using Amicon Ultra Centrifugal Filter 50 kDa (Merck Millipore) and the resulting suspension containing the aggregates were aliquoted and kept at -80 °C until use for the inoculations.

### Intracisternal injection

The mice were anesthetized with 5% isoflurane. To relieve pain, Carprofen (Rimadyl, 5 mg/kg, Pfizer) was injected subcutaneously. The mice were fixed on a small animal stereotaxic frame (Kopf Instruments) and the eyes were covered with ointment. During the procedure, the sedation was maintained by administrating 1.5-2% isoflurane via facemask and the body temperature was regulated using a TCAT-2LV temperature controller (Physitemp). The back of the neck was shaved and disinfected. An incision was made through the skin and fascia, and the muscle layers were retracted to expose the cisterna magna. A volume of 1 µl strain A homogenate or 5 µl hSOD1 strain A seeds, strain B seeds or control inoculate was injected into the cisterna magna using a 5 µl glass syringe with a custom 75RN (33G) needle (Hamilton). To minimize the backflow of CSF, the syringe was held in position for another 2 min after the injection. The skin incision was closed with tissue adhesive (Vetbond) and surgical clips (Reflex Autoclip System, 10 mm, AgnTho’s). After the surgery, the mice were rehydrated with a subcutaneous injection of saline solution and placed on a heating blanket for recovery from anesthesia.

### Monitoring of mice

The mice were examined and weighed weekly until disease onset and thereafter more frequently. The onset of disease was observed as weakness in a limb, judged by affected clinical grip strength, or/and flaccid or spastic splay of the hindlegs. The mice were considered terminally ill when severe forelimb weakness (as judged by loss of grip strength) was observed alone or in combination with eye infection. A weight loss after peek weight of -20% was also by itself considered terminally ill. In a few cases where hindlimb symptoms were more prominent (some control inoculated mice), they were considered terminally ill when both hindlimbs were fully or almost fully paralyzed.

### Excluded mice

Four mice were excluded and not reported in the Results or Discussion section. One mouse was euthanized pre-symptomatically because of wound unrelated to the surgical procedure and three did not recover from the surgical procedure.

### Tissue collection

At the time for tissue collection, the mice were anesthetized with a lethal dose of pentobarbital administered through an intraperitoneal injection. The mice dedicated to immunohistochemical analysis were perfused intracardially with saline followed by 10% buffered formalin and immersion fixed in the latter solution. The brain including the brainstem was coronally dissected into four slices of three millimeters thickness and the spinal cord was dissected into cervical, thoracic and lumbar sections. The tissues were dehydrated and embedded in paraffin for subsequent immunohistochemical analysis.

The mice dedicated to biochemical analysis were euthanized with a lethal dose of pentobarbital administered through an intraperitoneal injection. The brain was dissected from the skull, and the spinal cord was flushed out of the spinal column with saline using a syringe inserted at the caudal opening of the spinal column. The left and right side of the cervical, thoracic and lumbar spinal cord, cerebrum, brainstem and cerebellum were dissected and snap-frozen in -80 °C for subsequent analysis of structure and distribution of hSOD1 aggregates.

### Binary epitope mapping

The structure of the aggregates was analyzed using binary epitope mapping (BEM), as described previously [11]. Each tissue was homogenized in 25 volumes of ice-cold PBS containing 80 µl/ml Complete EDTA-free protease inhibitor cocktail (Roche Diagnostics), 1.8 mM EDTA and 1 mM DTT using Ultraturrax apparatus for 20 s per sample and sonicated for one min. The homogenates were then diluted in 20 volumes of PBS containing 80 µl/ml Complete, 1.8 mM EDTA and 1 mM DTT and 1% NP40, sonicated for 30 s per sample and centrifuged at 1000 x g for 10 min at 4 °C. The supernatant was serially diluted 1:1 in PBS and captured on a 0.2 μm cellulose acetate filter (Whatman) using a 96-well dotblot apparatus. After washing, the filter was blocked using 5% dry milk and 0.1% Tween for two hours. The filter was thereafter cut in slices and incubated with the eight anti-hSOD1 antibodies mentioned earlier at 4 °C overnight. The dotblot was developed using a HRP-conjugated goat anti-rabbit Ig antibody (Dako) and ECL Select (Cytiva). The intensities were recorded using a Chemidoc apparatus (Bio-Rad) and analyzed with Quantity One software (Bio-Rad).

To allow quantification of staining intensities, spinal homogenates from an end-stage hSOD1^G93A^ transgenic mouse treated as described above were used as a standard and set to 1. The hSOD1^G93A^ homogenate was serially diluted 1:1 and immunolabeled with the anti-hSOD1^57–72^ antibody on all filters. All homogenates labeled with all antibodies were quantified against this standard. This standard was chosen since the hSOD1^G93A^ mice are the most used ALS-model. To enhance appreciation of staining patterns in each homogenate, the staining intensities of the eight antibodies were normalized against the signal detected by the anti-hSOD1^57–72^ antibody within the same homogenate, which was set to 100%. The graphs for BEM were created using Prism (GraphPad) version 10.2.1.

### Immunohistochemistry

The formalin fixed paraffin embedded tissues were cut to 5 μm thick sections using a microtome and mounted on glass slides (Superfrost, Thermo scientific). Apart from the immunostains slides of all tissues were also stained using Hematoxylin/ Eosin. Thereafter, the tissues were immunostained according to the manufacturer’s recommendations using the automated stainer Discovery Ultra (Roche Diagnostics). The tissues were treated with either heat-induced antigen retrieval before incubation with the primary antibody anti-hSOD^131–153^ (0.96 µg/ml) or enzymatic antigen retrieval (Ventana Protease 1) before incubation with the primary antibody anti-hSOD^111–127^ (0.86 µg/ml). The primary antibodies were detected using anti-rabbit secondary antibodies conjugated with horseradish peroxidase (Omni-Map, Roche Diagnostics) and visualized using DAB staining (ChromoMap DAB detection kit, Roche Diagnostics). The sections were counterstained with Hematoxylin II (Roche Diagnostics), washed, dehydrated and finally mounted with Pertex (DakoCytomation). Images were obtained using Olympus BX61 and the Olympus cellSens Standard software version 3.2.

### Statistics

Statistical analyses were performed using an Independent-Samples Mann-Whitney U test in the IBM SPSS Statistics software version 29.0.1.0. A P-value of 0.05 or less was considered statistically significant.

## Results

### Intracisternal injection of strain A and B seeds induces premature fatal ALS-like disease

To investigate whether CSF constitutes a route for the spread of SOD1-ALS, we injected hSOD1 aggregates into the cisterna magna of 100 days old asymptomatic hSOD1^G85R^ Tg mice. The hSOD1^G85R^ Tg model has a long symptom-free period followed by a late middle-age onset of SOD1 aggregation and muscle paresis, which is an advantage for detecting effects of seeding. The homogenate and seeds used for inoculations were prepared from spinal cords of terminally ill hSOD1^G85R^ Tg, hSOD1^D90A^ Tg and non-Tg C57BL/6 mice.

**Fig. 1:**
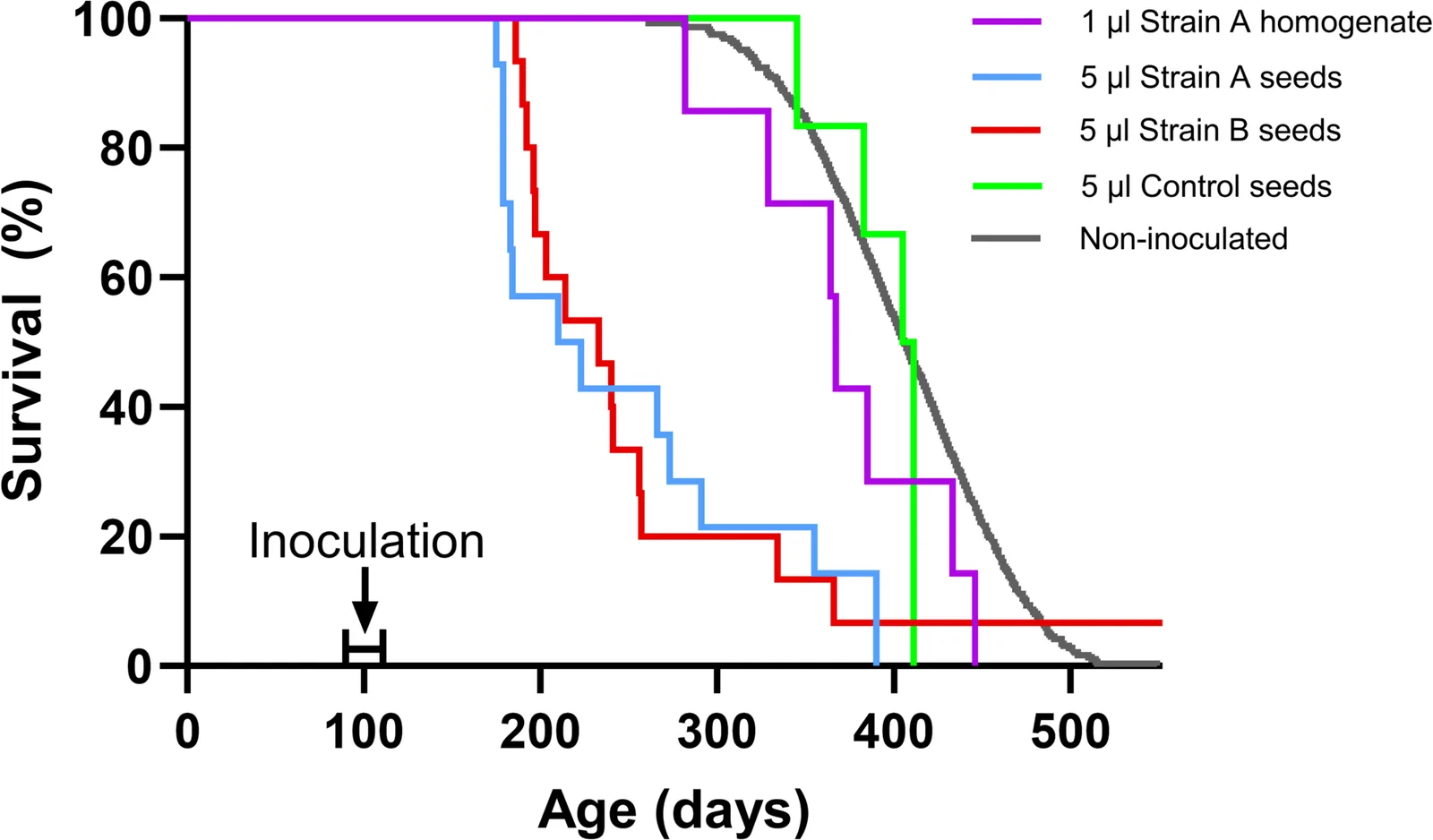
Intracisternal inoculation of strain A and B seeds induces premature fatal ALS-like disease. The Kaplan-Meier curves indicate the survival of hSOD1^G85R^ Tg mice inoculated with strain A seeds (blue, *n* = 14), strain B seeds (red, *n* = 15), strain A homogenate (purple, *n* = 7) or control sample (green, *n* = 6) into the cisterna magna. Survival data of non-inoculated mice are included as a reference (grey, *n* = 287)

Inititally, 1 µl of strain A homogenate (concentration of SOD1 aggregates: 1.6 µg/ml) was injected into the cisterna magna of 100 days old asymptomatic hSOD1^G85R^ Tg mice. This amount of SOD1 aggregates has previously been sufficient to initiate templated seeding when injected into the lumbar spinal cord of the same mouse model [27]. In our current study, there was no significant difference between the mice inoculated with 1 µl of strain A homogenate and non-inoculated mice (*p* = 0.147, Mann Whitney U-test) (Fig. 1).

Since the aggregates become diluted in the CSF following the inoculation into the cisterna magna, we tested whether a larger amount of hSOD1 aggregates would be sufficient for seeding. To do this, new seed preparations were made to concentrate molecules of high molecular weight. A volume of 5 µl seed preparation of strain A (~ 9 µg/ml hSOD1 aggregates), strain B (~ 13 µg/ml hSOD1 aggregates) or C57BL/6 control sample (hSOD1 aggregates not detectable) was injected into the cisterna magna of 100 days old asymptomatic hSOD1^G85R^ Tg mice. Mice inoculated with strain A or B showed the first signs of disease 87 days and 92 days after inoculation, respectively, which was approximately 150 days before control inoculated mice (Fig. 1; Table 1). Additionally, strain A and B inoculated mice reached terminal disease stage approximately 180 days before control inoculated mice (Fig. 1; Table 1). However, no difference in disease course was observed between strain A and B inoculated mice (Fig. 1; Table 1). Finally, the control inoculated mice did not differ in survival or phenotype from non-inoculated mice (Fig. 1).

**Table 1:** Development of pathology in inoculated mice

	Significance	Strain A seed inoculated	Strain B seed inoculated	Strain A homogenate inoculated	Control inoculated
		*n* = 11	*n* = 12	*n* = 7	*n* = 6
	*P*	Days	Days	Days	Days
Age at inoculation	0.721	106 ± 7^†^	110 ± 5^†^	106 ± 3^‡^	111 ± 1^‡^
Time to symptom onset	< 0.001	87 ± 32^†^	92 ± 23^†^	234 ± 52^‡^	243 ± 35^‡^
Disease duration	< 0.001	19 ± 11^†^	16 ± 13^†^	32 ± 16^‡^	40 ± 11^‡^
Age at fatal disease stage	< 0.001	213 ± 44^†^	217 ± 27^†^	372 ± 57^‡^	394 ± 26^‡^

Data represent the mean ± SD. P-values, extracted from Mann Whitney U test, represent the differences between mice inoculated with strain A or B seeds (†) and mice inoculated with strain A homogenate or controls (‡)

Time to symptom onset, disease duration and age at fatal disease stage were significantly lower in mice inoculated with strain A or B seeds compared to mice inoculated with strain A homogenate or control inoculated mice. In total, twenty-nine hSOD1^G85R^ Tg mice were inoculated with either strain A or strain B seeds. However, three animals in each of the two groups appeared to be outliers since their lifespans were similar to non-inoculated mice (see Fig. 1), suggesting failed inoculations. Additionally, one of them did not develop any ALS symptoms within expected timespan of a non-inoculated hSOD1^G85R^ Tg mouse and was sacrificed because of old age, it was thus considered to be non-Tg. The six outliers were therefore omitted from the statistical calculations in the table.

### Strain A and B seeds induce templated aggregation occurring along the neuroaxis

**Fig. 2:**
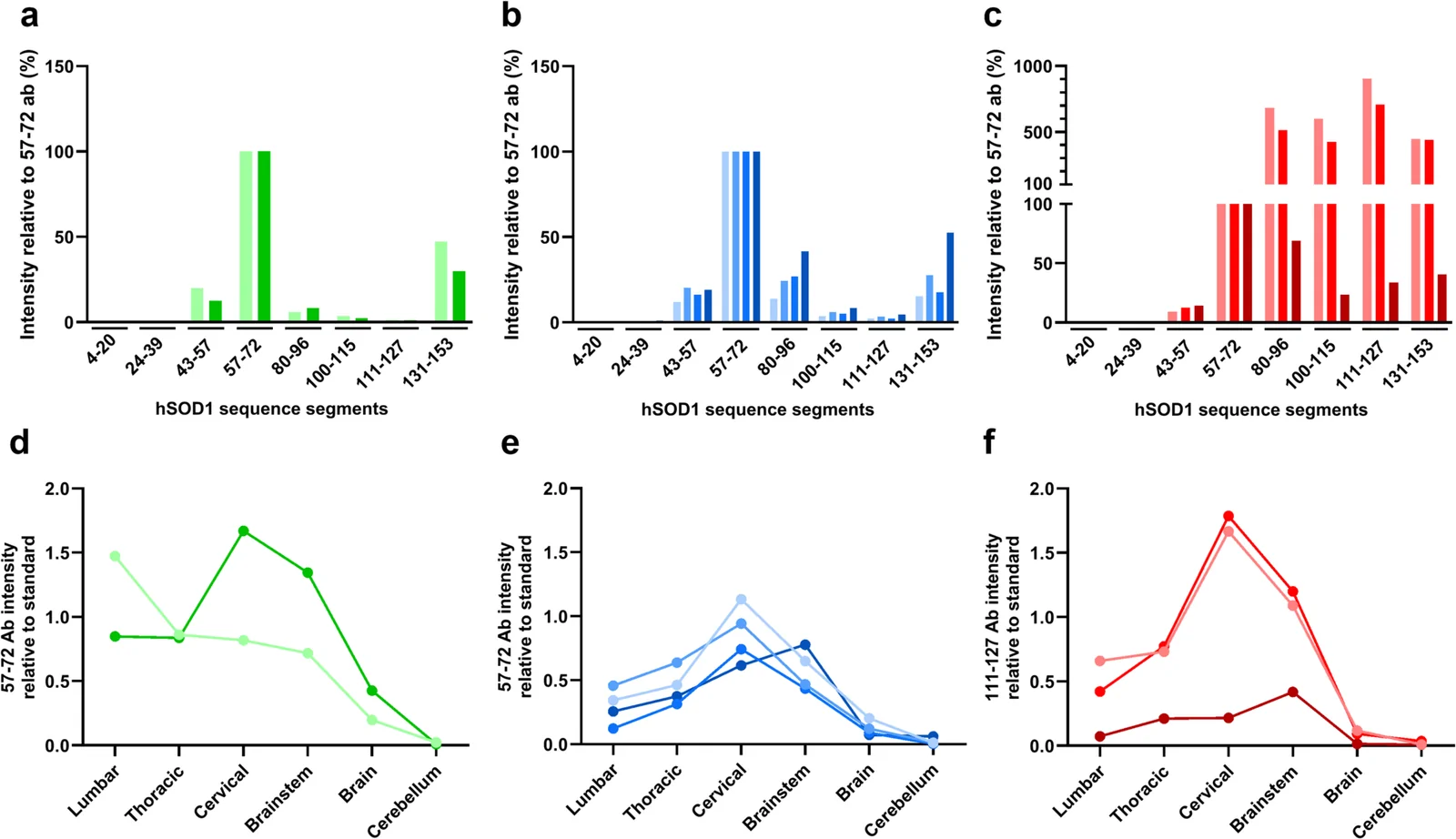
Strain A and B seeds induce templated aggregation spreading along the neuroaxis. Binary epitope mapping of cervical spinal cord (**a**–**c**) and quantitative epitope mapping along the neuroaxis (**d**–**f**) of terminally ill control inoculated mice (*n* = 2) (**a**,** d**); terminally ill strain A inoculated mice (*n* = 4) (**b**,** e**); and terminally ill strain B inoculated mice (*n* = 3) (**c**,** f**). Each color nuance represents an individual mouse. To facilitate the comparison of patterns, the staining intensities were normalized against the signal detected by the hSOD1^57–72^ Ab which was set to 100% (**a**–**c**). The hSOD1^57–72^ Ab and hSOD1^111–127^ Ab were used to analyze the distribution of strain A and strain B, respectively (**d-f**), and the Ab staining intensities were quantified using spinal homogenates from a hSOD1^G93A^ Tg mouse as a standard and set to 1

To investigate the structure of hSOD1 aggregates expressed by cisterna magna-injected mice at terminal disease stage, BEM was used. Strain A seed inoculated mice expressed a strain A pattern similar to the control inoculated mice (Fig. 2a, b). Two out of three strain B inoculated mice expressed a typical strain B pattern with the N-terminal folded into the fibril core and the rest protruding into the solvent in a disordered manner (Fig. 2b). The disordered 111–127 aa sequence in strain B aggregates was confirmed using histopathological immunolabeling (Supplementary Fig. 2). Note that strain B is not spontaneously formed in hSOD1^G85R^ Tg mice, indicating a templated prion-like spread of the seeds. Interestingly, one mouse showed a lower reactivity in the sequences towards the C-terminal end (Fig. 2c).

The distribution of hSOD1 aggregates along the neuroaxis at terminal disease stage was analyzed using the hSOD1^57–72^ Ab for strain A and hSOD^111–127^ for strain B. For the majority of both strain A and B inoculated mice, the highest level of aggregates was seen in the cervical spinal cord (Fig. 2e, f). However, for one mouse in each of the two groups, the highest level was observed in the brainstem. Corresponding with the aggregate distributions, the strain A and B inoculated mice showed predominently forelimb symptoms at the end stage of the disease. For one of the two control inoculated mice, the highest level of hSOD1 aggregates was seen in the lumbar spinal cord and for the other it was seen in the cervical spinal cord (Fig. 2d), corresponding to predominent hindlimb and forelimb symptoms, respectively.

Using H/E stained sections no granulocytes were seen in CNS tissue proper (single ones could be detected inside blood vessels but at a very low frequency well within the numbers seen in normal mice). No large clusters of round cells or activated microglia was detected anywhere. Thus, there is an absence of inflammation not only in the vicinity of the inoculation site but in all of the investigated areas. Reactive gliosis is seen as normally in hSOD1 ALS transgenic animals in affected areas especially in terminal animals. 

### Neuronal protein aggregation is accelerated in strain A and B inoculated mice

**Fig. 3:**
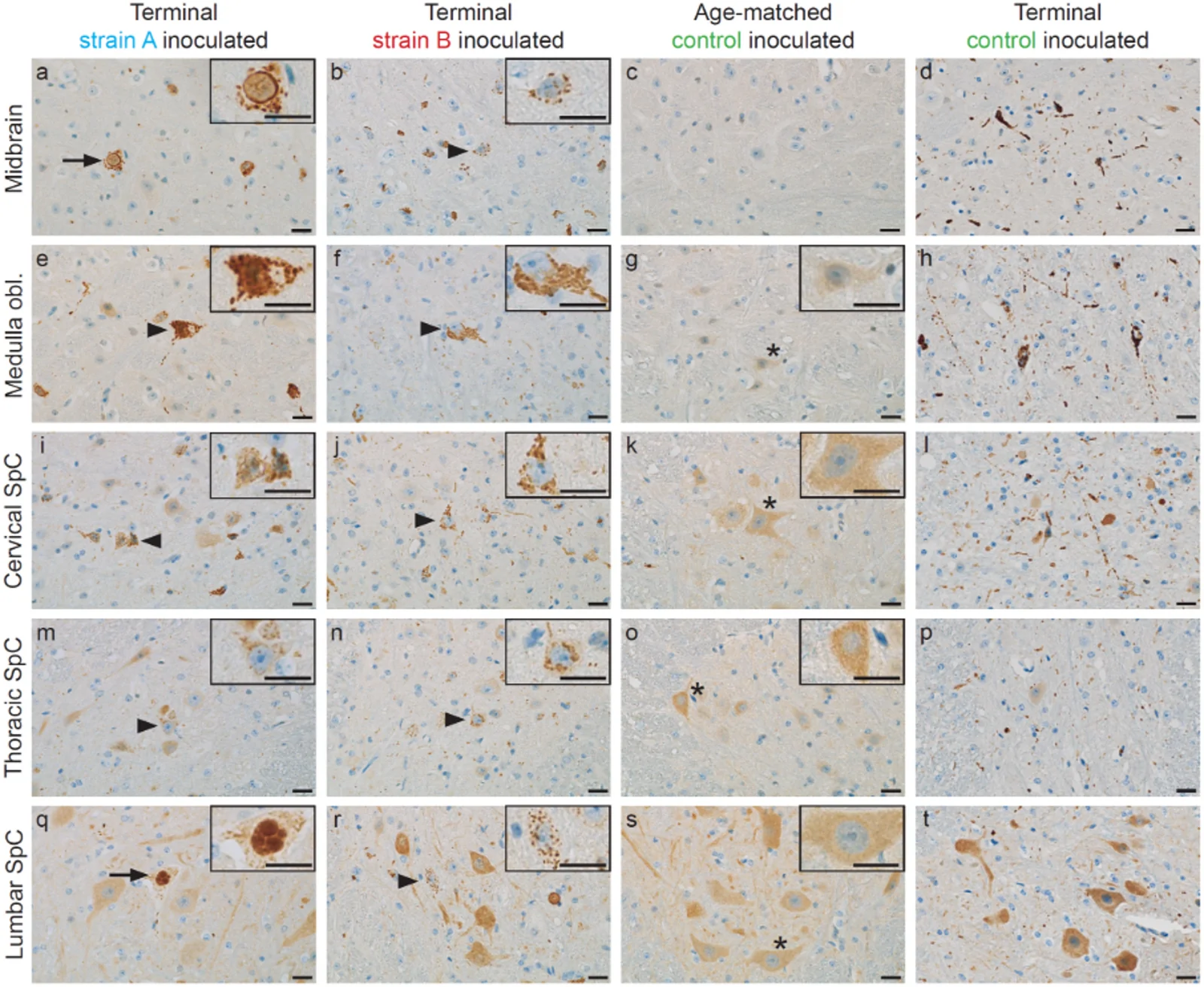
Histopathology of the ventral horn of hSOD1 strain A and B inoculated mice and control inoculated mice. The tissues were stained with a rabbit antibody targeting the aa sequence 131–153 of hSOD1. Terminal strain A (**a**,** e**,** i**,** m**,** q**) and strain B (**b**,** f**,** j**,** n**,** r**) inoculated mice show cytoplasmic condensed hSOD1 aggregates (arrowheads, high magnification insets) in neurons throughout the spinal cord and brainstem. *Strain A* inoculated mice also show perinuclear (**a**, arrow, high magnification inset) and nuclear (**q**, arrow, high magnification inset) staining of hSOD1. The age-matched control inoculated mice (about 250d) (**c**,** g**,** k**,** o**,** s**), show cytoplasmic granular staining of hSOD1 in neurons (asterisks, high magnification insets). At terminal disease stage (about 400 days), the control inoculated mice show cytoplasmic condensed hSOD1 aggregates in neurons (**d**,** h**,** l**,** p**,** t**). Scale bar: 20 μm

At terminal disease stage, the immunohistochemical analyses (Fig. 3, Suppl Fig. 4) revealed cytoplasmic condensed hSOD1 aggregates in neurons throughout the ventral horn of the spinal cord and brainstem in strain A (Fig. 3a, e, i, m, q) and strain B inoculated mice (Fig. 3b, f, j, n, r). The aggregates were more abundant in the brainstem and cervical spinal cord compared to thoracic and lumbar spinal cord which correspond to the symptom phenotypes and propagation data shown in Fig. 2e, f. In addition to the cytoplasmic aggregates, nuclear and perinuclear staining was also seen in the strain A inoculated mice (Fig. 3a, q).

In contrast, neurons of control inoculated mice which were age-matched to terminal strain A and B mice, showed an evenly distributed cytoplasmic staining of disordered SOD1 which appeared more pronounced in the lumbar spinal cord compared to superior levels of the CNS (Fig. 3c, g, k, o, s). The control inoculated mice later developed condensed hSOD1 aggregates which was seen at terminal disease stage (Fig. 3d, h, l, p, t). These findings show that the protein aggregation is accelerated in strain A and B inoculated mice compared to control inoculated mice.

## Discussion

The prion-like spreading mechanism of SOD1 and other proteins associated with neurodegenerative disease such as amyloid-β, α-synuclein and tau has recently been studied extensively, and the data are robustly supported by studies using different methodologies. However, mapping the transmission pathways utilized by these pathogenic protein assemblies in vivo is an ongoing progress. Emerging evidence suggests that CSF may play a role in the intercellular spread of proteinopathies [19, 32, 34, 40, 41]. In this report, we demonstrate for the first time that human SOD1 aggregates are capable of inducing templated aggregation and cause premature ALS while being transmitted from the CSF to the CNS.

Our study shows that injection of hSOD1 seeds into CSF of adult asymptmatic hSOD^G85R^ Tg mice induced premature ALS-like disease and shortened survival significantly compared to control inoculated and non-inoculated mice. The hSOD^G85R^ Tg mice show exponential aggregation late in the life in contrast to the hSOD^G93A^ Tg mice [12]. This gives a large window of opportunity to identify induced early aggregation. Additionally, we observed a prion-like spread of SOD1 aggregates appearing along the neuroaxis with strain A and B inoculated mice developing A and B aggregates, respectively, and the control inoculated mice expressed a late strain A pattern as expected from the endogenous hSOD1^G85R^ background. The highest amount of aggregates was found in the brainstem and cervical spinal cord which are next to the site of inoculation. Interestingly, the cerebrum and cerebellum, which are also close to the cisterna magna, contained much sparser amount of aggregates. This indicates that the brainstem and spinal cord are especially susceptible to SOD1 aggregation while the cerebellum and cerebrum are less vulnerable.

The G85R is destabilized and lacks the intrasubunit disulfide bond. However, the total level is low, about as low as total SOD1 levels in human CNS [42, 43]. Other transgenic mice with relatively stable mutants as hSOD1^G93A^ and the (wildtype-like) hSOD1^D90A^, contain much higher total levels of SOD1 in the CNS. They also contain subfractions that are destabilized [10, 44]. The amounts of destabilized SOD1 are similar in all three models, although is clearly highest in the hSOD1^G93A^ model. This fact, coupled with the long window of opportunity for seeing increase in aggregation in the hSOD1^G85R^ model early appearance (from before birth) of high molecular weight aggregates in the hSOD1^G93A^ model, makes the hSOD1^G85R^ model a much better experimental model [12]. In a newly published paper [45] we report effects of inoculation of seeds derived from human CNS tissue from ALS patients homozygous for the *D90A* mutation to hSOD1^G85R^ mice. Even with this stable mutant, we see prion-like spread.

Notably, the one strain A and one strain B inoculated mouse which expressed the highest levels of aggregates in the brainstem had already lost weight before observed disease onset and had an exceedingly short observed symptomatic disease course of one and two days, respectively, a phenotype resembling bulbar-onset human ALS. Interestingly, the strain B inoculated mouse with short observed disease course, also showed a different aggregate structure pattern with lower antibody intensity values in the sequences towards the C-terminal end, compared to the other strain B inoculated mice. When plotting the quantative BEM against the hSOD1^57–72^ Ab, normally used to analyze strain A aggregates, the mouse also showed higher levels of aggregates in the CNS, compared to the plot using the hSOD1^111–127^ Ab (Supplementary Fig. 1, purple). Even though the seeds used for strain B inoculations are derived from homozygous D90A mice with dominating strain B levels, strain A also co-exist in these mice, though in lower levels [11]. It therefore appears possible that in this mouse, the seed transmitted growth of both aggregate types, yielding a mixture of strain A and B in the CNS segments analyzed.

Moreover, SOD1 aggregation was observed to be accelerated in strain A and B inoculated mice compared with control inoculated mice (Fig. 3). This was demonstrated by condensed neuronal aggregates in terminal strain A and B inoculated mice while the age-matched control inoculated mice showed evenly distributed cytoplasmic staining similar to the histological appearance at disease onset shown in our previous study [12]. The presence of inclusions and accelerated aggregation following the intracisternal injection further confirms the ability of SOD1 to enter the CNS via CSF.

Similar to the findings in this study, shortened survival, prion-like spread along the neuroaxis and presence of neuronal cytoplasmic SOD1 inclusions was observed in previous studies when hSOD1 seeds or homogenates were inoculated into the lumbar spinal cord of the same mouse model [12, 27]. In those studies, injection of 1 µl homogenate was sufficient to initiate templated seeding in the CNS and the effect was still evident after injecting a 27-fold diluted homogenate, altough less pronounced [27]. The subarachnoid space in mice has a volume of around 37 µl [46] and a high CSF production rate of around 0.35 µl/min [47]. This bulk flow means that their total CSF voulme is replaced every 1.8 h. Altough the concentration of inoculated hSOD1 aggregates in our current study is higher than in our previuos experiments [12] and the levels of SOD1 previously measured in human CSF [36–38], the dilution effect may explain why a larger amount of hSOD1 seeds was needed to observe a seeding effect when injecting them into the CSF of the mice. However, if comparing the doses used presently, assuming av total mouse CSF volume of 37 µl [46] a cisternal inoculation of 5 µl seed preparation results in an estimated 7.4 (37/5) dilution. The seed preparation contained approximately 10 µg/mL (Suppl Table 1) yielding a final hSOD1 concentration of approximately 1.35 µg/mL in the CSF. These concentration far exceeds reported hSOD1 levels in the human CSF as it contains approximately 0.12 µg/mL hSOD1 [36, 37, 43]. Note that using a dose 1/5 of that here described we did not observe any effect of the survival within the survival times of mice.

Further evidence of a dilution effect in the present system is the fact that inoculation of the homogenate preparation which have a lesser SOD1 content than the other batches [Supplemental Table 1]), resulted in slower symtom onset and a longer survival (Fig. 1).

Additionally, the spread of inoculate following an injection into the cisterna magna has previously been demonstrated by intracisternal inoculation of 12 µl Trypan Blue. The inoculation results in a spread into the 4th ventricle as well as around the base of the brain and around cerebellum [46]. Although SOD1 seeds are of higher molecular weight than Tryptan Blue, it seems that an inoculation of 5 µl would spread into brain stem cisterns as well as into the 4th ventricle even if diluted.

In our previous study, where strain A and strain B were inoculated into the lumbar spinal cord, cytoplasmic SOD1 inclusions were found in neurons of strain A and B lumbar inoculated mice and distributed mainly throughout the spinal cord and brainstem with higher levels in the caudal segments [12]. Altough the highest levels of aggregates were seen more rostrally in our current study, the spinal cord and brainstem were still the most affected regions (Fig. 2e, f). Additionally, the ventral horn of the spinal cord was distinctly more affected by SOD1 aggregation compared to the dorsal horn in these studies (Supplemental Fig. 4) [12]. Collectively, these findings indicate that the locations of aggregation and intracellular morphology of SOD1 aggregates are similar despite different sites of injection/routes of administration, further suggesting certain areas and cellular compartments to be particularly susceptible to SOD1 aggregate formation than others.

With regard to the present design we could not discriminate between directional propagation or multifocal seeding from the CSF, which would be a question for future studies.

As reported earlier, we previously homogenized fresh (within 30 min after dissection) non-frozen murine tissues from the CNS and from the periphery in buffer containing a thiol-blocking agent to prevent artificial oxidation of the C57–C146 disulfide bond [38]. We found a marked enrichment of disordered mutant human SOD1 monomers in spinal cord compared to the brain and even less in the peripheral tissues [44, 48]. Such analyses cannot be made with human autopsy tissues, but the levels of several important autophagy factors have been found to be lower in the lamina IX of the ventral horn compared to other areas of the CNS [35]. Thus, insufficient control of SOD1 structure and aggregation could be important factors behind the vulnerability of motor areas to SOD1-linked neurotoxicity.

The phenotypes provoked by strain A and B have previously been reported to differ regarding disease progression rate [12]. However, no difference in disease progression was observed between mice intracisternally inoculated with strain A or strain B.

In this study, we used three batches of strain A prepared at different timepoints and, to our surprise, we observed a difference in survival between them (Supplementary Fig. 3); two being more potent than the third one. When combining the graphs of the different strain A batches, there is no significant difference between strain A and B. Interestingly, the hSOD1 protein content is nearly the same between the different batches of strain A (Supplementary Table 1) and there is no difference in survival when they are injected into the lumbar spinal cord (Supplementary Table 2). Although the reason for this finding remains elusive, one possibility for this variability might be the homogenizing and sonication step in the preparation. These are difficult to exactly control and might result in the high molecular weight aggregates being fragmented to different extents. However, it is an observation to take into consideration in future seeding studies.

In conclusion, we here demonstrate for the first time that SOD1 aggregates are capable of entering the CNS from the CSF where they induce premature ALS-like disease, spread along the neuroaxis in a prion-like manner and form cytoplasmic inclusions in neurons. The results demonstrate that CSF may constitute a route of spread of SOD1 associated ALS, indicating SOD1 in CSF to be a potential therapeutic target. Moreover, unraveling the transmission pathways utilized by pathological protein aggregates opens new avenues for preventing the spread of ALS and other neurodegenerative diseases. 

## Supplementary Information

Below is the link to the electronic supplementary material.

Supplementary Material 1.

## Data Availability

Data is available upon request.
