# Supplementary material for: Intracisternal injection of two human SOD1 aggregate strains induces templated aggregation and ALS in transgenic mouse model
Source: Acta Neuropathol Commun. 2026 Oct 27;14:225. doi: 10.1186/s40478-026-02473-x (PMC13715270; doi:10.1186/s40478-026-02473-x)
Supplement: Supplementary file 1 [file 40478_2026_2473_MOESM1_ESM.docx]

## Supplementary material

### Intracisternal Injection of SOD1 Aggregates Induces ALS Disease in Transgenic Mouse Model

Isabelle Sigfridsson^1^, Matthew Marklund^1^, Peter Munch Andersen^2^, Stefan L. Marklund^1^, Thomas Brännström^1
1^Department of Medical Biosciences, Umeå University, 901 87, Umeå, Sweden
^2^Department of Clinical Sciences, Neuroscience, Umeå University, 901 87, Umeå, Sweden

Correspondence to: Thomas Brännström
E-mail: [thomas.brannstrom@umu.se](mailto:thomas.brannstrom@umu.se)
Telephone: +46706343932

**Supplemental Tables**

Supplemental Table 1

| Seed | Mice used (n) | Total protein (µg/ml) | SOD1 (µg/ml) |
| --- | --- | --- | --- |
| Strain A seed I | G85R (10) | 6800 | 9.9 |
| Strain A seed II | G85R (12) | 5910 | 8.5 |
| Strain A seed III | G85R (11) | 4460 | 8.5 |
| Strain B seed | D90A (11) | 2480 | 13 |
| Strain A homogenate | G85R (9) | 2080 | 1.6 * |
| Control seed | C57BL/6 (4) | 7790 | 0 |

* The amount of SOD1 aggregates in the homogenate was calculated by ultracentrifugation and quantitative western blot as previously described (Leykam et al, 2025, J Neurochem).

Supplemental Table 2

| **Lumbar injection** | | | |  | **Intracisternal injection** | |  |
| --- | --- | --- | --- | --- | --- | --- | --- |
|  | **Strain A Batch 1** | **Strain A Batch 3** | **Significance** | **Strain A Batch 1** | **Strain A Batch 3** | **Strain A Batch 2** | **Significance** |
|  | *n = 2* | *n = 4* |  | *n = 5* | *n = 4* | *n = 2* |  |
|  | **Days** | **Days** | **P** | **Days** | **Days** | **Days** | **P** |
| **Age at inoculation** | 103 ± 2^†^ | 98 ± 3^†^ | 0.133 | 101 ± 5^‡^ | 114 ± 4^‡^ | 107 ± 0 | 0.016 |
| **Time to symptom onset** | 54 ± 6^†^ | 51 ± 6^†^ | 1.000 | 63 ± 6^‡^ | 119 ± 30^‡^ | 86 ± 23 | 0.016 |
| **Disease duration** | 8 ± 4^†^ | 19 ± 3^†^ | 0.133 | 16 ± 6^‡^ | 31 ± 5^‡^ | 5 ± 5 | 0.016 |
| **Age at fatal disease stage** | 164 ± 1^†^ | 167^†^ ± 6^†^ | 0.533 | 184 ± 3^‡^ | 263 ± 29^‡^ | 197 ± 18 | 0.016 |

Supplemental Table 2. Development of pathology in mice inoculated with different strain A batches into lumbar spinal cord or cisterna magna. Data represents the mean ± SD. P-values, extracted from Mann Whitney U test, represent the differences between mice inoculated with batch 1 and batch 3 into the lumbar spinal cord (†) and mice inoculated with batch 1 and batch 3 into the cisterna magna (‡). Time to symptom onset, disease duration and age at fatal disease stage were significantly shorter in mice inoculated with batch 1 in cisterna magna. No difference in disease progression was observed between the different batches when inoculated into the lumbar spinal cord. Batch 2 was not used in lumbar inoculations and therefore not included in the statistical comparison analysis.

### Supplemental Figures

**2.0**

**1.5**

**Ab intensity relative to standard**

**1.0**

**0.5**

**0.0**

**Lumbar**

**Thoracic**

**Cervical**

**Brainstem**

**Brain**

**Cerebellum**

Supplemental Fig. 1. Quantitative BEM of terminally ill strain B inoculated mice. Each color represents an individual mouse (n=3). Dotted lines represent staining intensities of hSOD1^111-127^ Ab and solid lines represent staining intensities of hSOD1^57-72^ Ab. The intensity values were quantified using spinal homogenates from a hSOD1^G93A^ Tg mouse as a standard and set to 1.

Supplemental Fig. 2. Histopathology of strain A and strain B inoculated mice immunolabeled with anti-hSOD1^111-127^ Ab. Aggregates of hSOD1 in the brainstem of terminal strain B inoculated mice show positive staining of the 111-127 aa sequence (a, b). Tissue derived from strain A inoculated mice showed much only faint staining of the hSOD1^111-127^ Ab. Scale bar: 20 µm.

**100**

**80**

**Survival (%)**

**60**

5 μl Strain A seeds (Batch 1) 5 μl Strain A seeds (Batch 2) 5 μl Strain A seeds (Batch 3) 5 μl Strain B seeds

**40**

**20**	Inoculation

**0**

**0	100	200	300	400	500**

# Age (days)

Supplemental Fig. 3. Intracisternal injection of different batches of strain A affects the progression of disease differently. Dark blue line indicates the survival of mice inoculated with strain A seeds from batch 1 (n=5); mid blue line indicates the survival of mice inoculated with strain A seeds from batch 2 (n=2); turquois line indicates the survival of mice inoculated with strain A seeds from batch 3 (n=4); red line indicates the survival of mice inoculated with strain B (n=11).

Supplemental Fig 4. Histopathology of hSOD1 immunolabeling in dorsal horn of the spinal cord. The tissues were stained with a rabbit antibody targeting the aa sequence 131-153 of hSOD1. Strain A inoculated mice (a, e, i), strain B inoculated mice (b, f, j), age-matched control inoculated mice (c, g, k) and terminal control inoculated mice (d, h, l) show sparse amount of hSOD1 staining in the dorsal horn of the spinal cord. Scale bar: 20 µm.
